# Musculoskeletal pain is common in competitive gaming: a cross-sectional study among Danish esports athletes

**DOI:** 10.1136/bmjsem-2020-000799

**Published:** 2020-08-28

**Authors:** Line Lindberg, Simon Bay Nielsen, Mads Damgaard, Ole Rolskov Sloth, Michael Skovdal Rathleff, Christian Lund Straszek

**Affiliations:** 1 Department of Physiotherapy, University College of Northern Denmark, Aalborg, Denmark; 2 Center for General Practice, Aalborg University, Aalborg, Denmark; 3 Department of Health Science and Technology, Aalborg Universitet, Aalborg, Denmark

**Keywords:** Athlete, Physical activity, Sport, Muscle damage/injuries

## Abstract

**Objectives:**

The interest for competitive esports is growing. Little is known regarding musculoskeletal (MSK) pain among esports athletes. We aimed to investigate (1) the prevalence of MSK pain, (2) the association between MSK pain and esports-related training volume and (3) the association between MSK pain and physical activity levels.

**Methods:**

Athletes aged 15–35 years who participated in structured esports through a computer-based game were eligible for inclusion. Participant demographics, hours/week spent on esports, self-report MSK pain sites, pain frequency, sleep, care-seeking behaviour and physical activity levels were collected through online questionnaires. The primary outcome was any MSK pain in the body during the previous week.

**Results:**

Of 188 included athletes, 42.6% reported MSK pain. The most common pain site was the back (31.3%). Athletes with MSK pain participated in significantly less esports training compared with athletes without MSK pain (mean difference −5.6 hours/week; 95% CI −10.6 to −0.7, p=0.035). There was no significant difference in physical activity levels between groups (mean difference 81.1 metabolic equivalent of task-minutes/week; 95% CI −1266.9 to 1429.1, p=0.906).

**Conclusion:**

Back pain is common among esports athletes. Athletes with MSK pain participated in less esports training compared with those without pain, suggesting a potentially negative effect of pain on esports participation.

## INTRODUCTION

Esports is the fastest growing sport in Denmark, which grows 133% in memberships per year.^1^ Esports is defined as ‘a form of sports where the primary aspects of the sport are facilitated by electronic systems; the input of players and teams as well as the output of the E-sport system are mediated by human-computer interference’.^2^ Esports is considered sedentary in nature due to the lack of bodily movement but may impose significant demands on the body due to rapid and continuous movements of the fingers and concentration demands.^3 4^ Esports athletes practise for 5.5–10 hours daily prior to competition.^5^ It is unclear how such high work demands may affect musculoskeletal (MSK) pain in esports athletes and subsequently the athlete’s performance. DiFrancisco-Donoghue *et al* recently performed the first study among a small group of 65 college esports athletes. They discovered that 41% suffered from back or neck pain and 36% reported wrist pain.^5^ As MSK pain is associated with frequent use of healthcare services and analgesics, and sleep impairment,^6^ these findings warrant further larger studies on MSK pain health status among this emerging sport.

The aims of this study were to (1) investigate the prevalence of MSK pain among esports athletes, (2) assess if training volume among athletes with MSK pain was different from athletes without MSK pain and (3) investigate if physical activity levels among athletes with MSK pain were different from athletes without MSK pain.

## METHOD

This study was conducted at the Department of Physiotherapy at University College of Northern Denmark, Aalborg, Denmark. The study was preregistered on www.ClinicalTrials.gov (NCT03910517). Participants received oral and written study-information before providing written informed consent.

### Eligibility criteria

We aimed to include 200 participants, which represented 10% of all registered athletes in Denmark in 2017.^7^ At the time, approximately 60% of the registered esports athletes were between the ages of 15 and 35.^1^ Therefore, athletes were eligible for inclusion if they

- Were 15–35 years of age.
- Participated in structured esports (defined as training with a coach).
- Primarily engaged in esports through a computer-based game.

### Distribution of questionnaire

The questionnaire was developed and distributed via SurveyXact. Prior to initiation of the study, the questionnaire was pilot tested and evaluated regarding time to complete, relevance and comprehensibility among esports athletes.

### Participant characteristics and outcome

Athletes were asked to provide name, email, phone number, age, gender, height, weight, smoking status, where they participate in esports (eg, community-based or educational institution) and their primary game.

#### Primary and secondary outcome

The primary outcome was ‘any MSK pain during the previous week’ (answer being yes/no). Participants were also asked about primary and/or secondary pain sites. Worst pain at the primary pain site during the previous week was assessed with an 11-point numeric pain rating scale (0=no pain; 10=worst possible pain). Pain frequency was also assessed. To investigate the impact of their pain, athletes were asked if their participation in esports was impaired due to MSK pain. A list of secondary outcomes regarding esports-related training volume, physical activity levels, care-seeking behaviour and sleep is presented in table 1.

**Table 1:** Secondary outcomes

Esports-related training volume Hours of structured esports/week. Hours of unstructured esports/week. Total hours of esports/week (sum of weekly structured and unstructured esports participation).
Physical activity levels Danish version of the International Physical Activity Questionnaire short form.^8^
Use of healthcare services and analgesics Care sought from a healthcare professional during the past 3 months because of MSK pain. Current use analgesics and type.
Sleep patterns Average hours of sleep during the night. Trouble falling asleep. Interrupted sleep during the night. Feeling tired in the morning.

MSK, musculoskeletal.

### Data analysis

All statistical analyses were conducted in IBM SPSS Version 26. To investigate if esports-related training volume differed among athletes with or without MSK pain, we used total esports-related training volume (total hours of structured esports/week+total hours of unstructured esports/week). To assess if physical activity levels were different between athletes with and without MSK pain, we used the total metabolic equivalent of task (MET) minutes per week scores from the International Physical Activity Questionnaire(IPAQ) short form. Data from 34 participants (13/34 with MSK pain during the previous week) were not included in the analysis regarding physical activity levels due to inadequate reporting in relation to the IPAQ short from. As such, data from 154 participants were analysed. Due to the exclusion of these participants from the primary analysis, a sensitivity analysis was conducted to test if excluding these participants would change the result of the primary analysis. For comparisons, we used an independent sample t-test or the Wilcoxon rank-sum test depending on the distribution of data. A p value <0.05 was considered statistically significant.

## RESULTS

**Figure 1:**
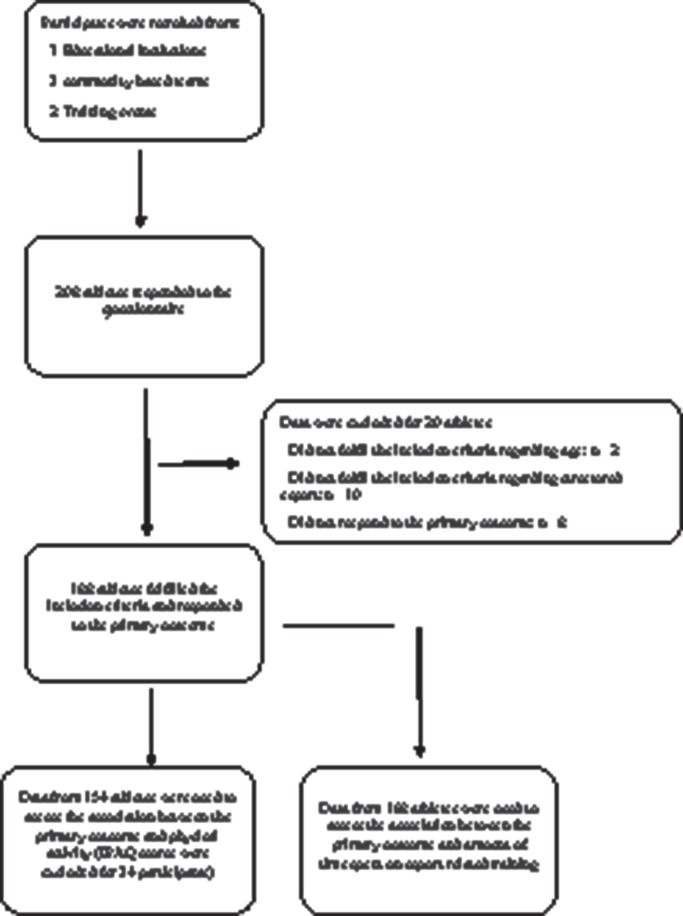
Flow chart.

From 27 March to 26 April 2019, we recruited a total of 208 esports athletes from two esports training events, three community-based teams and seven educational institutions who offered esports to their students in the Northern part of Jutland, Denmark. In total, responses from 188 esports athletes were included for analysis (figure 1). See table 2 for additional data regarding participant characteristics and table 3 for findings on the use of healthcare service and analgesics and sleep patterns.

**Table 2:** Participant characteristics for all participants (n=188), for those with any MSK pain (n=80) and those with no MSK pain (n=108)

Variables	All (n=188)	Any MSK pain during previous week (n=80)	No MSK pain during previous week (n=108)
Age (years)	17.1 (2.3)	17.1 (2.5)	17 (2.1)
Sex, n males (%)	184 (97.9)	76 (95)	108 (100)
Height (m)	1.8 (0.08)	1.8 (0.1)	1.8 (0.07)
Weight (kg)	73.3 (17.0)	75 (20.7)	71.9 (13.7)
BMI (weight/height^2^)	22.3 (4.8)	22.6 (5.4)	22 (4.4)
Smoking status (n reporting ‘yes’ (%))	18 (9.6)	10 (12.5)	8 (7.4)
Primary game, n (%)			
Counter-Strike: Global Offensive	109 (58)	42 (52.5)	67 (62)
League of Legends	51 (27.1)	25 (31.3)	26 (24.1)
Others	28 (14.9)	13 (16.3)	15 (13.9)
Esports participation n (%) - Educational institution - Community-based team - Pro-team	146 (77.7) 30 (16) 4 (2.1)	65 (81.3) 9 (11.3) 1 (1.3)	81 (75) 21 (19.4) 3 (2.8)
Others	8 (4.3)	5 (6.3)	3 (2.8)
Hours of structured esports/week (95% CI)	6.9 (6.3 to 7.5)	6.5 (5.6 to 7.5)	7.2 (6.4 to 7.9)
Hours of unstructured esports/week (95% CI)	17.3 (15.0 to 19.5)	14.4 (11.4 to 17.4)	19.4 (16.2 to 22.6)
Total hours of esports/week (95% CI)	24.2 (21.7 to 26.7)	20.9 (17.6 to 24.3)	26.6 (23.1 to 30.1)

Data is presented with mean and standard deviation unless otherwise specified. MSK, musculoskeletal.

**Table 3:** Use of healthcare services and analgesics, and sleep patterns

Variables	All (n=187)	Any MSK pain during previous week (n=80)	No MSK pain during previous week (n=107)
Have you sought care for any MSK complaint during the previous 3 months? n (%) - Yes, with a chiropractor - Yes, with my general practitioner - Yes, with a physiotherapist - Yes, with an orthopaedic surgeon - Yes, with a rheumatologist	8 (4.3) 7 (3.7) 11 (5.9) 0 (0) 0 (0)	8 (10) 5 (6.3) 8 (10) 0 (0) 0 (0)	0 (0) 2 (1.9) 3 (2.8) 0 (0) 0 (0)
Analgesics utilisation (n reporting ‘yes’ (%))	14 (7.5)	13 (16.3)	1 (0.9)
Type of analgesics, n (%) - Paracetamol - NSAID - Opioids - Others - Don’t know	9 (4.8) 6 (3.2) 1 (0.5) 2 (1.1) 3 (1.6)	9 (11.3) 6 (7.5) 1 (1.3) 2 (2.5) 2 (2.5)	0 (0) 0 (0) 0 (0) 0 (0) 1 (0.9)
Sleep			
Hours of sleep during the night (95% CI)	7.4 (7.2 to 7.6)	7.3 (7.0 to 7.5)	7.6 (7.4 to 7.8)
Trouble falling asleep % (95% CI) - Yes, most nights - Yes, some nights - No, not at all - Don’t know	10.6 (6.9 to 15.9) 48.4 (41.2 to 55.5) 38.8 (32.0 to 46.0) 2.1 (0.7 to 5.5)	16.2 (9.6 to 26.1) 50.0 (39.0 to 61.9) 31.2 (21.9 to 42.3) 2.5 (0.6 to 9.6)	6.4 (3.0 to 13.0) 47.2 (37.9 to 56.7) 44.4 (35.2 to 54.0) 1.8 (0.4 to 7.2)
Waking several times/nights % (95% CI) - Yes, most nights - Yes, some nights - No, not at all - Don’t know	3.1 (1.4 to 6.9) 23.4 (17.8 to 30.0) 71.2 (64.3 to 77.3) 2.1 (0.7 to 5.5)	5.0 (1.8 to 12.7) 27.5 (18.7 to 38.4) 65.0 (53.8 to 74.7) 2.5 (0.6 to 9.6)	1.8 (0.4 to 7.2) 20.3 (13.7 to 29.1) 75.9 (66.8 to 83.1) 1.8 (0.4 to 7.2)
Trouble sleeping through the night % (95% CI) - Yes, most nights - Yes, some nights - No, not at all - Don’t know	2.6 (1.1 to 6.2) 19.6 (14.5 to 26.0) 75.5 (68.8 to 81.1) 2.1 (0.7 to 5.5)	3.7 (1.2 to 11.1) 26.2 (17.6 to 37.0) 66.2 (55.1 to 75.8) 3.7 (1.1 to 11.1)	1.8 (0.4 to 7.2) 14.8 (9.2 to 22.9) 82.4 (73.9 to 88.5) 0.9 (0.1 to 6.4)
Waking feeling tired % (95% CI) - Yes, most mornings - Yes, some mornings - No, not at all - Don’t know	52.6 (45.4 to 59.7) 40.9 (34.0 to 48.1) 5.8 (3.2 to 10.3) 0.5 (0.0 to 3.7)	62.5 (51.3 to 72.4) 33.7 (24.1 to 44.8) 3.7 (1.1 to 11.1) 0 (0 to 0)	45.3 (36.1 to 54.9) 46.2 (37.0 to 55.8) 7.4 (3.7 to 14.2) 0.9 (0.1 to 6.4)

Data are reported as percentages with 95% CIs unless otherwise specified. Data from 187 in the ‘all’ category, 80 in the ‘Any MSK pain during the previous week’ category and from 107 in the ‘No MSK pain during previous week’ category (data missing from one athlete). MSK, musculoskeletal; NSAID, non-steroidal anti-inflammatory drugs.

### Musculoskeletal pain: prevalence, distribution and intensity

Of the 188 included esports athletes, 42.6% (80 athletes) reported MSK pain within the previous week with the back (31.3%), neck (11.3%) and shoulders (11.3%) being the most common pain sites. Thirty-two per cent experienced MSK pain at one site, 27% at two sites and 9% reported pain at three sites. The median number of pain sites was 2 (range 1–13), and the mean pain intensity was 4 (SD 1.8). Of those who experienced pain during the previous week, 6.25% had pain which limited their participation in esports-related activities (see online supplemental material S1 for additional data on pain distribution and frequency).

10.1136/bmjsem-2020-000799.supp1 Supplementary data

### Association between esports-related training volume and MSK pain

The average weekly esports-related training volume was significantly lower in the group who reported MSK pain during the previous week (20.9±15.1 hours) compared with the group with no MSK pain (26±18.5 hours) (mean difference −5.6 hours/week; 95% CI −10.6 to −0.7, p=0.027).

### Association between physical activity levels and MSK pain

The average weekly physical activity levels were not significantly lower in the group who reported MSK pain during the previous week (3722.4±3667.3 MET-minutes) compared with the group with no MSK pain (3641.3±4563.1 MET-minutes) (mean difference 81.1 MET-minutes/week; 95% CI −1266.9 to 1429.1, p=0.906). The sensitivity analysis supports these results.

## DISCUSSION

### MSK pain in relation to esports training volume and physical activity levels

We found a significant association between MSK pain and esports-related training volume. Those with MSK pain had 6 hours less esports training per week. This suggests that esports participation may be impaired among athletes with MSK pain as their weekly training volume was significantly lower compared with athletes without MSK pain. There was little difference in prevalence of MSK pain between the primary games played as nearly half of those who played Counter-Strike: Global Offensive and League of Legends reported MSK pain (table 2). There was no difference in physical activity levels between athletes with and without MSK pain indicating that MSK pain did not affect physical activity levels.

### Comparison with previous findings

The prevalence of MSK pain in our study was similar to DiFrancisco-Donoghue *et al* ^5^ who found that one in three college esports athletes suffered from back or neck. The population prevalence of back pain among Danish adolescents is 24.1% for both boys and girls, and 19.4% for boys alone.^9^ This indicates a higher prevalence of back pain among esports athletes compared with the background population. DiFrancisco-Donoghue *et al* ^5^ found a higher prevalence of both wrist (36%) and hand pain (30%) compared with the current study (wrist; 6.25% and hand/fingers; 5%). This difference could be explained by the large difference in esports training volume between the two studies (38–70 hours/week vs 24 hours/week in the current study).^5^

A cross-sectional study among 2000 office-based employees found a high prevalence of MSK pain in head/neck (42%), lower back (34%) and shoulders (16%).^10^ Back pain was most common among employees who were under 30 years of age and those who rated their workstation ergonomics as poor.^10^ The authors suggested the age-difference was due to higher computer interaction among younger employees compared with the senior staff.^10^ However, this raises the questions if comfort during esports participation may be associated with pain and if sitting ergonomics is a potential cause of the high prevalence of MSK pain among esports athletes with high levels of computer interaction.

On average, the esports athletes in this study used ≈3700 MET-minutes/week. These findings are in line with a study among virtual football players, which found that 73% of the study population reached 3000 MET-minutes/week.^11^ It is likely that most of these athletes were exposed to physical activity through physical education class, which could explain the relatively high weekly MET-minute scores.

### Limitations

We did not include a control group in this cross-sectional study. Only four participants were females most likely reflecting that a minority of competitive esports athletes are females.^1^ The prevalence of MSK pain was therefore compared indirectly to DiFrancisco-Donoghue *et al* ^5^ and Rathleff *et al.* ^9^ As the current study has a cross-sectional design, it is not possible to draw conclusions regarding causality (if participating in esports leads to MSK pain or vice versa). Self-reported measures of physical activity are susceptible to reporting bias,^12^ and therefore, the average MET-minutes per week could be overestimated.

### Implications for future research

One in 14 esports athletes experience MSK pain severe enough to affect esports participation, which underpins the need to develop sport-specific management strategies. The field within esports health is still emerging, consequently providing limited evidence to inform specific management strategies. DiFrancisco-Donoghue *et al* provide an example of how esports athletes could manage their health in collaboration with a variety of health professionals including physiotherapists, athletic trainers and exercise specialist.^5^ As only data from cross-sectional studies are available at present, future observational studies and intervention studies should be of longitudinal design to investigate exposure and outcome relationships.

**What are the new findings?**

- Musculoskeletal pain is prevalent in esports with 4 in every 10 athletes reporting pain.
- The most prevalent pain sites were the back (31.3%), neck (11.3%) and shoulders (11.3%).
- Athletes with musculoskeletal pain participated in significantly less esports-related training suggesting that musculoskeletal pain may have a negative effect on esports participation.
