# Supplementary material for: Musculoskeletal pain is common in competitive gaming: a cross-sectional study among Danish esports athletes
Source: BMJ Open Sport Exerc Med. 2020 Aug 28;6(1):000799. doi: 10.1136/bmjsem-2020-000799 (PMC7876625; doi:10.1136/bmjsem-2020-000799)
Supplement: Supplementary data [file bmjsem-2020-000799supp001.pdf]

Supplementary S1 Pain distribution and frequency

Variables	Any MSK pain during previous week (n=80)
**Pain sites, n (%)**	
**- Back**	**25 (31.3%)**
**- Neck**	**9 (11.3%)**
**- Shoulder**	**9 (11.3%)**
**- Head**	**7 (8.8%)**
**- Forarm**	**6 (7.5%)**
**- Wrist**	**5 (6.3%)**
**- Hand/fingers**	**4 (5.0%)**
**- Knee**	**4 (5.0%)**
**- Thigh**	**3 (3.8%)**
**- Ankle**	**3 (3.8%)**
**- Shinbone**	**1 (1.3%)**
**- Upperarm**	**1 (1.3%)**
**- Chest**	**1 (1.3%)**
**- Hip/groin**	**1 (1.3%)**
**- Foot/toes**	**1 (1.3%)**
**- Stomach**	**0 (0%)**
**- Elbow**	**0 (0%)**
**Pain frequency**	
**- Daily**	**23 (28.75%)**
**- Several times per week**	**11 (13.75%)**
**- Weekly**	**22 (27.5%)**
**- Monthly**	**14 (17.5%)**
**-Seldom**	**10 (12.5%)**
